# Socket seal surgery techniques in the esthetic zone: a systematic review with meta-analysis and trial sequential analysis of randomized clinical trials

**DOI:** 10.1186/s40729-021-00294-2

**Published:** 2021-02-22

**Authors:** Andrea López-Pacheco, David Soto-Peñaloza, Mayra Gómez, David Peñarrocha-Oltra, Marco Antonio Alarcón

**Affiliations:** 1 PerioImplant Research Group UPCH, Academic Department of Clinical Stomatology, Cayetano Heredia Peruvian University, Lima, Peru; 2 Oral Surgery Unit, Department of Stomatology, Valencia University Medical and Dental School, Valencia, Spain; 3 Academic Department of Clinical Stomatology, Section of Oral Implantology, Cayetano Heredia Peruvian University, Lima, Peru

**Keywords:** Tooth socket, Humans, Tooth extraction, Alveolar ridge augmentation

## Abstract

**Background:**

The socket seal surgery (SSS) technique is a common alternative for the management of the post-extraction sockets that requires a primary closure of the wound to promote proper regeneration and ridge preservation.

**Objective:**

To learn about the effect of different SSS techniques on alveolar ridge preservation

**Material and methods:**

Two independent and calibrated reviewers conducted an electronic search in PubMed, Cochrane, and Web of Science for randomized clinical trials (RCT) published up to June 2020. The evaluation of the risk of bias in the included studies was carried out following the Cochrane manual for interventions of systematic reviews, version 5.1.0. A meta-analysis of ridge width changes at − 1, − 3, and − 5 mm cutoff points from bone crest was conducted using a random-effects model. The risk of types I and II errors against accrued data was appraised obtaining the required information size using a trial sequential analysis package (TSA).

**Results:**

A total of 135 sockets located in the esthetic zone were evaluated with a minimum of a 3-month follow-up after tooth extraction in 6 RCTs. The evaluated SSS techniques were free gingival graft (FGG), collagen matrix (CM), collagen sponge (CS), acellular dermal matrix (ADM), and polytetrafluoroethylene membrane (PTFEm). The FGG in sockets without bone filling showed significant results in preserving both buccal and lingual bone height (− 1.42 mm in the experimental group versus − 0.01 in the control group). The comparison of CM and FGG with bone filling did not show clinical differences in terms of dimensional bone changes. No clinical differences were found in either width or gingival thickness when comparing CM and CS. The meta-analyses of RW changes comparing CM versus FGG showed no significant differences, but a trend for lessening horizontal reduction at − 1, − 3, and − 5 mm in favor of FGG. The TSA showed that accrued data did not reach the required information size, and more evidence is required for clinical significance inferences.

**Conclusions:**

There are several predictable SSS techniques to improve clinical results in ridge preservation. More clinical studies in the form of clinical trials are required to demonstrate the superiority of one technique over another.

**Supplementary Information:**

The online version contains supplementary material available at 10.1186/s40729-021-00294-2.

## Introduction

The esthetics in oral rehabilitation with dental implants represents a challenge in meeting the expectations and satisfaction of patients [1–3]. Many techniques seek to reduce bone resorption and subsequent soft tissue contour after dental extraction [4, 5], such as guided bone regeneration, connective tissue graft, and socket shield technique, among others. Guided bone regeneration performed to preserve the alveolar ridge usually requires a primary closure of the wound to promote proper regeneration and avoid contamination of the grafts [6, 7]. It involves making incisions and lifting a flap that may reduce the blood supply, and cause a marginal recession at the adjacent teeth, defective papillae, loss of keratinized mucosa, increased postoperative pain, and swelling in patients [7–9]. These may have more impact on the anterior maxilla, where the anatomic features such as the labial plate are usually thinner and the esthetic outcomes are challenging.

An alternative for the management of post-extraction sockets is “socket seal surgery” (SSS). This technique was first described by Landsberg and Bichacho [10]. It can be defined as a procedure that, through soft tissue grafts or biomaterials, can seal the socket, complementing the guided bone regeneration or acting alone to preserve the soft tissues, thereby preventing its collapse [11].

The SSS has achieved favorable results in various clinical studies [11–15], reaching values of more than 50% graft integration after 6 weeks [12]; it further achieves a pleasing and esthetically acceptable color [13]. This technique begins taking a circular-shape epithelial tissue graft from the palate, usually with a punch, of a diameter similar to the alveolus to be sealed. The thickness of the epithelial graft is on average 2 mm [14]. The graft is sutured carefully with 6 to 10 stitches (ideally 6–0 gauge) [14]. It is recommended that, before suturing, the edge of the socket epithelium is revived to achieve adequate adaptation and vascularization of the graft. Although it is a predictable technique, some authors experienced partial or total necrosis of the graft [11, 15].

Currently, alternative materials to the palate graft are used, such as the collagen matrix (CM), which avoids a second surgical area, reduces postoperative morbidity, and has satisfactory results in terms of esthetics and less scarring [16]. Likewise, other materials reported in the literature are collagen sponge (CS) [17], collagen membrane [18, 19], acellular dermal matrix (ADM) [20], and polytetrafluoroethylene membrane (PTFEm) [21], also reporting satisfactory results.

To our knowledge, this is the first review to focus solely on the effect of SSS on alveolar ridge preservation (ARP). This systematic review aims to evaluate radiographic and clinical dimensional changes of SSS techniques intended to preserve the alveolar ridge in post-extractive sockets.

## Material and methods

### Development of a protocol and registration

This systematic review was registered in the PROSPERO database under number CRD42018094314 and was written following the PRISMA (Preferred Reporting Items for Systematic Reviews and Meta-Analyses) statement [22]*.* AMSTAR 2 checklist was followed to ensure the quality and transparency of the search [23].

### Focused question

What is the effect of different socket seal surgery techniques on alveolar ridge preservation in the esthetic zone?

### Study population, type of intervention and comparisons, and outcome measurements

Only RCTs were included in the analysis. PICO elements were used for ordinate reporting of information [24]*.*

#### Population

Healthy individuals over 18 years old who had undergone any type of ARP and SSS following permanent single root tooth extraction in the maxilla. The minimum follow-up time was 3 months.

#### Intervention

Studies reporting on ARP including socket-sealing techniques.

#### Comparison

All possible comparisons among the included SSS materials were considered as long as the compared techniques had the same bone filler.

#### Outcome

Studies reporting at least one of the following measurement methods:

Primary outcome: alveolar ridge width (RW) changes in millimeters at different cutoffs from the alveolar bone crest.

Secondary outcome: alveolar ridge height of the buccal plate (RH) changes in millimeters and clinical changes in soft tissues.

### Search strategy

The search strategy involves both electronic and manual searches. Electronic searches were performed in three databases: The National Library of Medicine (MEDLINE via PubMed), the Cochrane Central Register of Controlled Trials (CENTRAL), and Web of Science. The search strategy included terms related to the intervention and used the following combinations of keywords: ((((((((tooth extraction) OR socket) OR tooth removal) OR alveoli)) OR immediate placement) OR postextraction)) AND ((((((((((free gingival punch graft) OR free gingival graft) OR porcine collagen matrix) OR autogenous soft tissue graft) OR socket seal) OR collagen matrix seal) OR collagen sponge) OR subepithelial connective tissue)) AND ((((tooth extraction) OR socket) OR tooth removal) OR alveoli)). The results were limited to human studies. Also, an electronic screening of grey literature through Literature Report [25] and OpenGrey databases [26], as well as the consulting of references list of included studies, was conducted to detect potential eligible titles. The following journals were also screened up to June 2020: Clinical Oral Implants Research, Clinical Implant Dentistry and Related Research, European Journal of Oral Implantology, Implant Dentistry, International Journal of Oral and Maxillofacial Implants, International Journal of Periodontics and Restorative Dentistry, Journal of Clinical Periodontology, Journal of Dental Research, Journal of Oral and Maxillofacial Surgery, Journal of Periodontology. A manual search was also made in the bibliographies of the articles included. Only articles in English were included.

### Eligibility criteria for study inclusion/exclusion

#### Inclusion criteria

- Studies on humans
- Patients needing a single root tooth extraction between maxillary premolars, who had undergone any type of ARP with SSS
- Flapless extraction
- Reporting at least one of the following measurement methods: clinical measurement, three-dimensional radiographic evaluation, or histological examination
- Randomized controlled trials

#### Exclusion criteria

- Studies on medically compromised patients (e.g., systemic diseases)
- General contraindications to implant surgery: untreated periodontitis, severe bruxism or clenching, immunosuppression, previous history of irradiation of the head and neck area, uncontrolled diabetes, heavy smoker (> 10 cigarettes/day), poor oral hygiene and motivation, current or past treatment with bisphosphonates, substance abuse (alcohol, drugs), psychiatric disorders.
- Socket sealing with a coronally advanced flap
- Studies using different bone filling materials per group
- Studies that compare alveolar ridge preservation techniques with bone graft versus spontaneous healing.
- Studies with the inability to complete the follow-up.

### Screening methods and data extraction

It was conducted independently and in duplicate by two reviewers (A.L.P and M.G). According to selection criteria, titles and abstracts of search results were screened, and potential articles, or those with insufficient data to make a clear decision, were analyzed in full text for the eligibility criteria. Disagreements are resolved by discussion and consultation with a third author (M.A.A). The reasons for exclusion at this or subsequent stages were recorded. The level of agreement between reviewers against title eligibility was done using kappa scores (Cohen’s ĸ coefficient) and interpreted according to Landis and Koch scale [27].

The following data are extracted in predefined Excel spreadsheets by two authors (A.L.P, M.G) and considering: author, year, country, reporting of a priori sample size estimation, sample size (sites), socket seal surgery approach, type of bone graft material, socket integrity, method of dimension measurement, follow-up, radiographic and clinical outcome measurements (alveolar ridge width, alveolar ridge height of the buccal plate, and buccal gingival thickness), study setting, and funding sources. The data extraction was ascertained for adequacy by a third author (M.A.A); disagreements were solved by consensus.

### Quality assessment

Assessment of the risk of bias was carried out by two reviewers (A.L.P, MG) independently. The assessment was carried out in duplicate and based on guidance from a modification of the Cochrane tool [28] used to evaluate the methodological quality of the included studies.

The following six parameters—random sequence generation, allocation concealment, blinding of participants and personnel, blinding of outcome assessment, incomplete outcome data, and selective reporting—were evaluated as low risk, moderate risk, or high risk of bias.

### Data analysis and synthesis of the results

Statistical data handling was performed by one author (D.S.P.). Random-effects meta-analysis is conducted for the primary outcome (ridge width changes) with at least 6 months follow-up after SSS. Forest plots were created to illustrate the effects of the meta-analysis results. The effect size between test and control groups are summarized as mean difference (MD) in millimeters, and its respective 95% confidence intervals (CI).

Because the results of ridge preservation procedures might be affected by either clinical (e.g., age, sex, surgery, clinical expertise) and methodological aspects inherent to clinical trials conduction, a Sidik-Jonkman (Hartung-Knapp-Sidik-Jonkman) random effects model is carried out because it provides adequate type I error rates [29]. This approach is more robust to changes in the heterogeneity variance estimates, especially in meta-analyses that contain few studies [30]. Between-study heterogeneity was visually inspected in the forest plots and by calculating the τ2 (absolute heterogeneity) and the *I*^2^ statistics (relative heterogeneity), and the corresponding nullity statistical *Q* test was calculated. The *I*^2^ index defines the proportion of total variability in the result explained by heterogeneity, but no chance. Heterogeneity was roughly categorized as low, moderate, and high to *I*^2^ values of 25%, 50%, and 75% [31]. Publication bias was investigated by visual inspection of the funnel plot and employing the Egger test only if at least 10 studies are included. In the case of high heterogeneity, a sensitivity analysis is conducted to test the robustness of estimates excluding risk populations or effect modifiers. A two-sided level of significance of 5% (*α* = 0.05) was established. The Stata/SE version 16.1 for Mac (StataCorp LP, College Station, TX, USA) was used for quantitative synthesis.

### Additional analysis

The meta-analytic output is tested for the propensity of type I (false positives) and type II (false genitives) statistical errors and to analyze the power of available evidence using the Trial Sequential Analysis (TSA) open-access software (Trial Sequential Analysis v0.9 β, Copenhagen Trial Unit, Center for Clinical Intervention Research, Denmark). A type 1 error of 5% and a power of 80% (type 2 error = 80%) were set to calculate trial sequential monitoring boundaries, futility boundaries, and the required information size (RIS). A “model variance-based” approach is defined for the inconsistency correction and *I*^2^ values established according to meta-analytic data output if the heterogeneity observed is zero; a lower inconsistency (*I*^2^ = 25%) is assumed for RIS estimation based on a random-effects model as previously reported [32]. The anticipated mean difference between intervention groups is setting for an expected minimal biological plausible difference (lower bound of 95% CI) of − 0.18, − 0.33, and − 0.38 mm between DBBM and DBBM-C, for horizontal ridge preservation at 1, 3, and 5 mm from the bone crest [33]. A graphical evaluation was performed to determine whether the *Z* curve (showing the treatment effect) crossed either monitoring or futility boundaries and to obtain the RIS threshold.

## Results

### Data collection

**Fig. 1:**
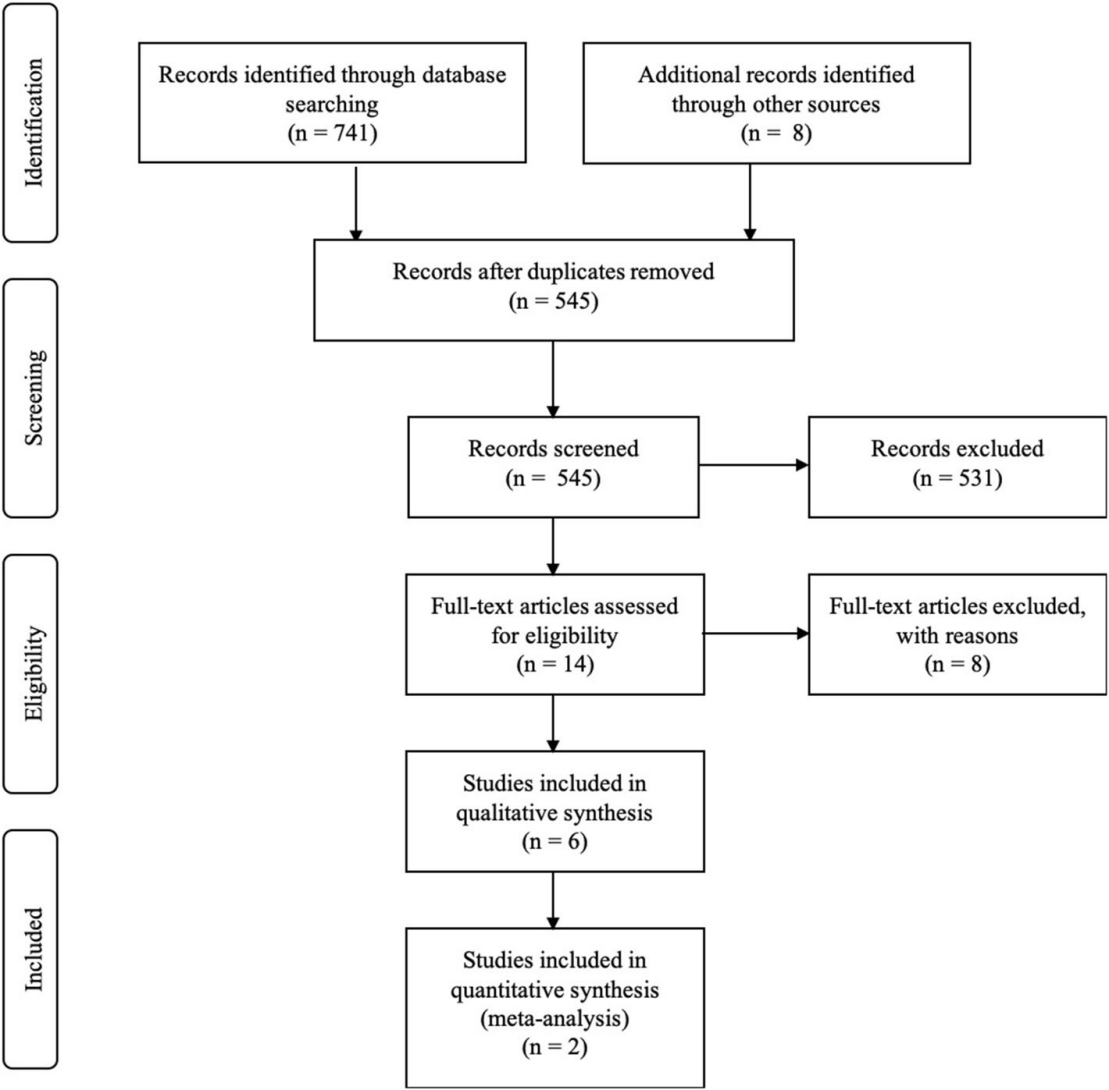
PRISMA flowchart of selection process

The initial search found a total of 741 records in the electronic and manual searches. After removal of duplicates and the title and abstract screening, a total of 14 articles remained for full-text assessment (Fig. 1). Six studies were finally included for qualitative analysis [34–39] and two for quantitative analysis [35, 37]. The reviewers showed an almost perfect level of agreement (*k* = 0.92). The most common reason for exclusion was those studies that compare different bone filling materials per group. The excluded papers and the reasons for exclusion are listed in Table 1, and the main characteristics of the included studies are summarized in Table 2.

**Table 1:** Studies excluded with reasons

Author	Year	Title	Reason for exclusion
Araujo and Lindhe [4]	2015	Ridge alterations following grafting of fresh extraction sockets in man. A randomized clinical trial.	Compare alveolar ridge preservation with bone graft versus spontaneous healing
Ávila-Ortiz et al.	2020	Efficacy of alveolar ridge preservation: a randomized controlled trial	Compare alveolar ridge preservation with bone graft versus spontaneous healing
Cardaropoli et al. [5]	2012	Socket preservation using bovine bone mineral and collagen membrane: a randomized controlled clinical trial with histologic analysis	Compare alveolar ridge preservation with bone graft versus spontaneous healing
Hu et al.	2009	Clinical study of tissue preservation of extraction socket with bio-oss collagen and acellular dermal matrix.	Compare alveolar ridge preservation with bone graft versus spontaneous healing
Neiva et al.	2011	Analysis of tissue neogenesis in extraction sockets treated with guided bone regeneration: clinical, histologic, and micro-CT.	Socket sealing with coronally advanced flap.
Oghli et al.	2010	Ridge preservation following tooth extraction: a comparison between atraumatic extraction and socket seal surgery.	Compare different bone filler per group
Park et al.	2016	The hidden X suture: a technical note on a novel suture technique for alveolar ridge preservation	Includes molar area
Sadatmansouri et al.	2013	Comparison of dPTFE and FGG for socket bone augmentation: a clinical and histological study	Different language
Thalmair et al. [40]	2013	Dimensional alterations of extraction sites after different alveolar ridge preservation techniques - a volumetric study.	Compare different bone filler per group

**Table 2:** Characteristics of the included studies

Author	Year	Country	A priori sample size estimation	Sample Size (sites)	Socket seal surgery approach		Type of bone graft material	Socket integrity	Method of dimensions measurement	Follow-up	Outcome measurements						Setting	Funding sources
					Intervention	Comparison					Radiographic				Clinical			
											Width	***p***	Height	***p***	Buccal gingival thickness	***p***		
Fotek et al. [34]	2009	USA	No	18 (18)	T: acellular dermal matrix	C: PTFE membrane	MBA	NR	Clinical	4 m	NR		NR	T^−5^, − 0.06 ± 0.24 mm C^−5^, − 0.17 ± 0.24 mm		0.28	University	BioHorizons and Osteogenics Biomedical
Jung et al. [35]	2013	Switzerland	Yes	20 (20)	T: collagen matrix	C: Free gingival graft	DBBM-C	At least 50% of the buccal bone wall	CBCT	6 m	T^−1^, − 1.2 ± 0.8 mm C^-1^, − 1.4 ± 1.0 mm	0.9	T, 0.0 ± 1.2.mm C, + 1.2 ± 2.9 mm	0.44	NR		University	University of Zurich and Geistlich Pharma AG
											T^−3^, − 0.6 ± 0.6 mm C^−3^, − 0.6 ± 0.5 mm	0.77						
											T^−5^, − 0.1 ± 0.2 mm C^−5^: − 0.6 ± 0.9 mm	0.09						
Karaca et al. [36]	2015	Turkey	No	10 (20)	T: free gingival graft	C: spontaneous healing	NONE	NR	CBCT	3 m	T^0^, − 0.99 ± 0.8 mm C^0^, − 1.22 ± 1.0 mm	0.24^a^	T, + 0.6 ± 1.2.mm C, − 1.03 ± 2.9 mm	0.03^a^	NR		University	The authors declare no sources of funding.
Meloni et al. [37]	2015	Italy	Yes	30 (30)	T: collagen matrix	C: soft tissue punch graft	DBB	Fenestration or dehiscence ≥3 mm on CBCT	CBCT	5 m	T^−1^, − 0.67 ± 0.31 mm C^−1^, − 0.54 ± 0.25 mm	0.34	T, − 1.47 ± 0.48 mm C, − 1.60 ± 0.69 mm	0.67	NR		Private practice	The authors declare no sources of funding.
											T^−3^, − 0.91 ± 0.26 mm C^−3^, − 0.83 ± 0.38 mm	0.61						
											T^−5^, − 0.31 ± 0.18 mm C^−5^, − 0.26 ± 0.17 mm	0.55						
Natto et al. [38]	2017	USA	Yes	28 (28)	T: collagen matrix	C: collagen sponge	FDBA	Presence of buccal plate	Clinical + CBCT	4 m	T^−4^, − 1.47 ± 1.29 mm C^−4^, − 1.21 ± 1.22 mm	0.49	T, − 0.79 ± 3.07 mm C, − 0.30 ± 1.09 mm	0.58	T^−4^, + 0.59 ± 1.28 mm C^−4^, + 0.90 ± 0.90 mm	0.46	University	Tufts University School of Dental Medicine
											T^−7^, − 0.96 ± 0.97 mm C^−7^, − 0.90 ± 1.02 mm	0.77			T^−7^, − 0.23 ± 1.42 mm C^−7^,+ 0.47 ± 1.16 mm	0.17		
											T^−10^, − 0.57 ± 0.99 mm C^−10^, − 0.54 ± 0.95 mm	0.84			T^−10^, − 0.13 ± 1.07 mm C^−10^, + 0.05 ± 1.62 mm	0.73		
Schneider et al. [39]	2014	Switzerland	Yes	19 (19)	T: collagen matrix	C: free gingival graft	DBBM-C	At least 50% of the buccal bone wall	Clinical	6 m	NR	NR			T, − 1.15 ± 0.50 mm C, − 1.16 ± 0.68 mm	1	University	University of Zurich and Geistlich Pharma AG

T and C superscript indicates the apical reference point from alveolar bone crest in mm (0, − 1, − 2, − 3, − 4, − 5, − 7, − 10 mm)

*MBA* Mineralized bone allograft, *DBBM-C* Demineralized bovine bone mineral + collagen matrix, *DBB* Demineralized bovine bone, *FDBA* Freeze-dried bone allograft, *CBCT* Cone beam computed tomography

^a^Statistically significant

### Characteristics of included studies

#### Study design and population

A total of 135 sockets in the esthetic zone were evaluated in 6 RCTs. Five studies presented a parallel design [34, 35, 37–39], and one presented a split mouth design [36]. Only two studies perform an a priori power calculation [37, 38]. The study population ranged from 18 to 30 individuals.

#### Type of intervention and biomaterials

A flapless extraction approach was performed in all studies. Different techniques were described for SSS: FGG [35–37, 39], CM [35, 37–39], CS [38], ADM [34], and PTFEm [32]. In three of the included studies, bone xenografts were the material chosen [35, 37, 39], two studies used bone allografts [34, 38], and only one study did not use any bone graft material [36].

### Methods of measurement

Changes in the primary outcomes were assessed by clinical and radiographic examinations. Bone changes in width and height were examined in four studies using CBCT [35–38]. Clinical measurement was reported in three studies [34, 38, 39]; one study evaluated the ridge width using digital models [39]; one study recorded the soft tissue thickness using stents [34]. Moreover, only one study registers the width of keratinized tissue band at the baseline and the endpoint of the investigation [38].

### Qualitative synthesis of study outcomes

The height of the buccal and lingual crest was analyzed in a split-mouth study [36] comparing sockets that had healed spontaneously with those treated with FGG without additional bone graft. After 3 months of healing, the control sockets had lost height in the buccal crestal bone − 1.42 mm; however, the height in the buccal crestal bone was preserved at the test sites − 0.01 mm. This difference between the two groups was statistically significant (*p* ≤ 0.05). In contrast, both the control and test groups lost width in the buccal and lingual crestal bone; the difference between the control and test groups was not statistically significant (*p* ≥ 0.05).

Socket seal surgery using FGG or CM was compared by CBCT analysis in two studies [35, 37]. After 5 [37] and 6 months [35], they found width changes at 1 mm below the crest, between − 0.54 and − 1.4 mm respectively, for sockets sealed with FGG, and − 0.67 and − 1.2 mm respectively in sockets sealed with a CM. This difference between the two groups was not statistically significant (*p* > 0.05).

Natto et al. [38] evaluated the results of CM seal in similar hard and soft tissue remodeling compared to that with CS used as barriers at 4 months following ARP, in combination with freeze-dried bone allograft. They found that reduction in coronal ridge width (− 1.21 mm CM and − 1.47 mm CS) and vertical buccal bone resorption (− 0.30 mm CMS and − 0.79 mm CS) was not statistically significant. A slight increase in buccal gingival thickness at the coronal part was observed in both groups (0.9 mm CM and 0.5 mm CS). The width of keratinized tissue decreased by an average of 0.08 mm in both groups.

The use of resorbable and non-resorbable membranes in ARP was compared by Fotek et al. [34]. The PTFEm exfoliated prematurely by 28 days, whereas the ADM appeared to be incorporated into the tissues. After 4 months, they found ridge width changes between − 0.06 ± 0.24 mm for sockets sealed with ADM and − 0.17 ± 0.24 mm in sockets sealed with a PTFEm. No statistically significant difference was observed between the two treatment modalities (*p* = 0.280).

### Risk of bias of included studies

**Fig. 2:**
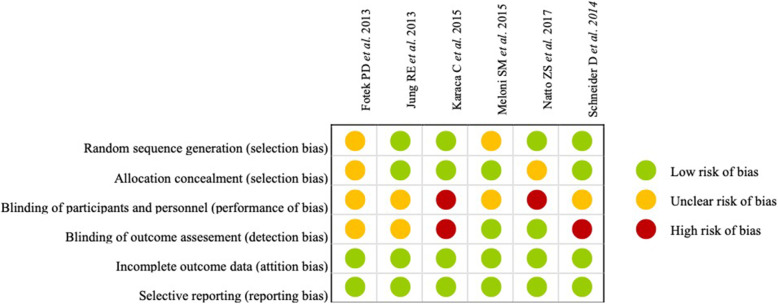
Risk of bias of the included studies

Blinding of participants and personnel was not comprehensively reported, resulting in an unclear risk of bias for these domains in all studies. However, random sequence generation, allocation concealment, attrition, and reporting were of less concern in most studies (Fig. 2).

### Quantitative summary–meta-analysis

**Fig. 3:**
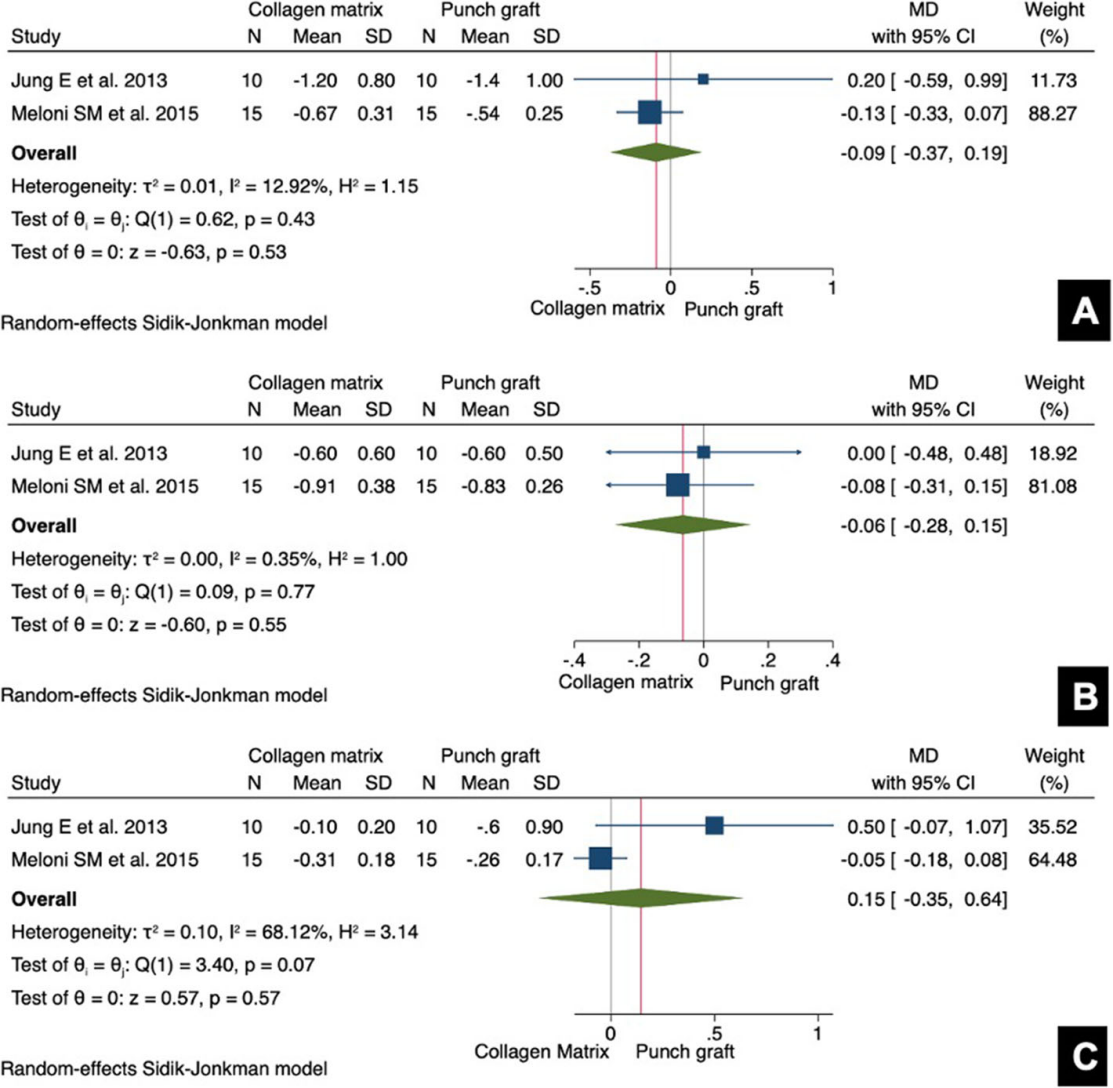
Forest plots of meta-analyses evaluating alveolar ridge width changes at different cutoff points from the alveolar bone crest. **a** RW at − 1 mm. **b** RW at − 3 mm. **c** RW at − 5 mm

Meta-analysis was able only for the primary outcome of the review. Only 2 studies allow a statistical comparison for RW reduction at − 1, − 3, and − 5 mm from bone crest after 6 months of socket sealing surgery [35, 37]. The results of meta-analyses are depicted visually throughout forest plots (Fig. 3). It was observed that mean differences of RW reduction did not reach statistical significance among data subsets (*p* ≥ 0.05). The horizontal bone reduction tended to be more accentuated within collagen matrix group at 1 mm (MD − 0.09; 95% CI [− 0.37; 0.19]; *I*^2^ = 13%) and 3 mm (MD − 0.06; 95% CI [− 0.28; 0.15]; *I*^2^ = 0%) cutoff points from alveolar bone crest (Fig. 3a, b). These findings occur in a context of low heterogeneity. The analysis at 5 mm from bone crest showed an increase of RW dimension in favor of FGG group (MD 0.15; 95% CI [− 0.35; 0.64]; *I*^2^ = 68%), but in a context of high heterogeneity. Egger’s test for publication bias is not applicable due to the lack of information (≤ 10 studies); indeed, it is visually explored using funnel plots, which showed a slight asymmetry. Though, these observations must be considered as merely exploratory and interpreted with caution (Additional file 1).

### Results of TSA

**Fig. 4:**
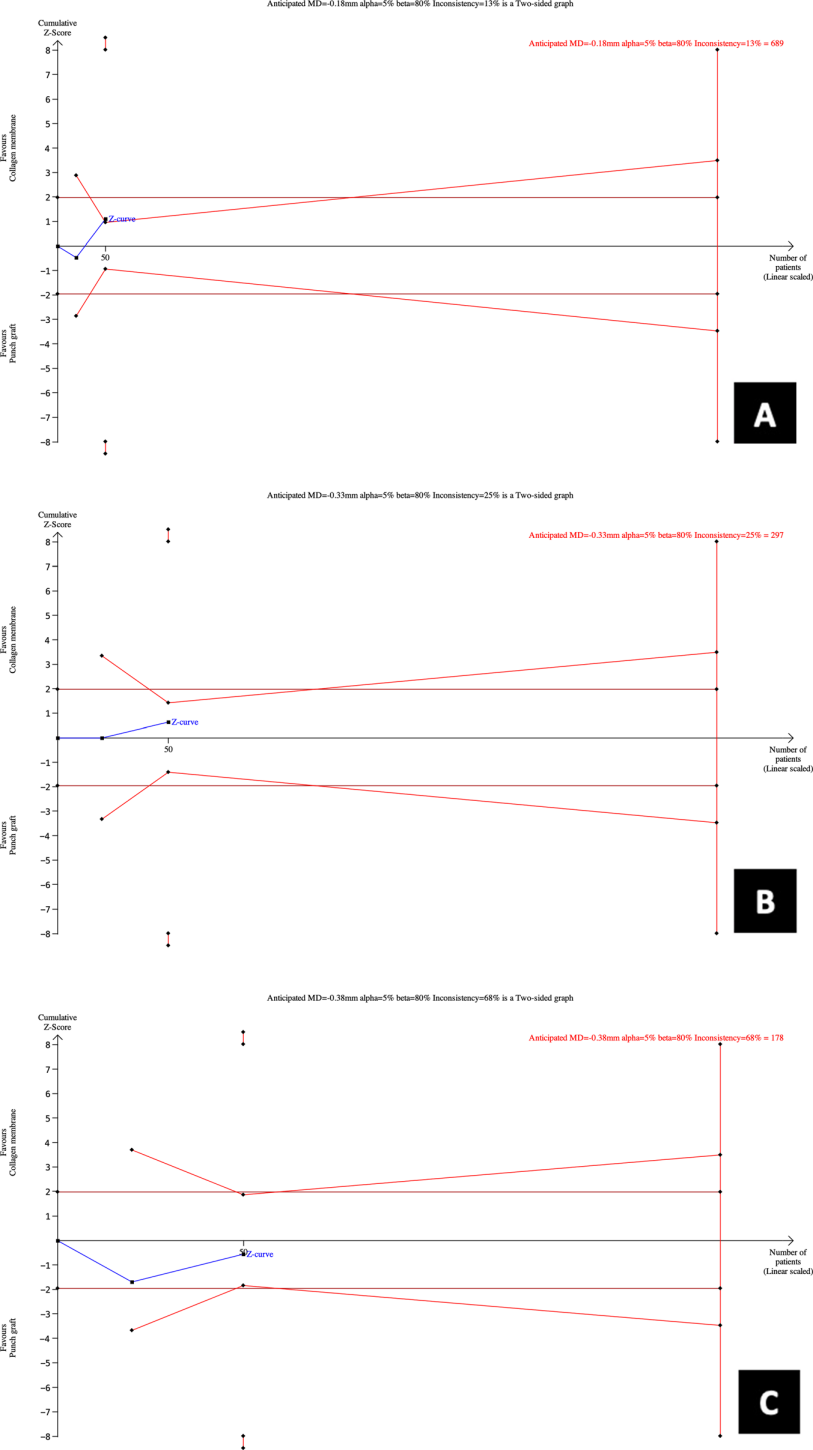
Trial sequential analysis for required information size estimations. **a** RW − 1 mm. **b** RW – 3. **c** RW − 5 mm

It was observed how the *z* curve does not cross the statistical significance threshold (brown lines) nor the monitoring boundaries’ (red lines). A sample of 297 grafted sites (individuals) is required for an anticipated horizontal ridge width reduction of − 0.33 mm at 3 mm, for an alpha error (0.05%), and power (1-beta = 0.20%) in a two-tailed statistical test. This required information size is adjusted, assuming a low heterogeneity (*I*^2^ = 25%) because the observed inconsistency in this meta-analysis was zero. The RIS obtained surpassed the accrued data in the present meta-analysis (Fig. 4b).

The RIS was estimated for the ridge width reduction at − 1 and − 5 mm from the bone crest. The RIS estimation was set according to anticipated mean differences as mentioned above, and it was observed that accrued meta-analytical data for RW reduction was not reached (Fig. 4b, c). Thus, more studies are warranted to determine which alveolar socket seal technique provides better results on ridge preservation, due to the sparse data available to make clinical significance inferences.

## Discussion

The present work aimed to assess the socket-sealing surgery techniques intended to preserve the alveolar ridge in post-extractive sockets. To date, despite being a routine clinical procedure, there are few qualified RCTs available to evaluate the effect of different SSS techniques for ARP. After literature screening, six studies were included in qualitative synthesis, from which only two RCTs [35, 37] allowed its statistical comparison for ridge width reduction at 6 months, and considering different cutoffs points (− 1, − 3, and − 5 mm) from the bone crest. The SSS techniques found in this systematic review were described as FGG, CM, CS, ADM, and PTFEm providing effective results in the esthetic zone [34–39].

Regarding methodological quality, most studies showed a parallel arm design [34, 35, 37–39], except a study with split-mouth approach [36]. The most critical methodological concerns are related to the blinding of participants and personnel. Note to mention, blinding of participants in surgical trials are challenging to perform, and evidence suggests that bias associated with lack of blinding and lack of concealment may be higher in trials with subjective outcomes [41].

In short, this review showed that the SSS techniques enhance the ARP outcomes at both alveolar bone dimensional changes. Notwithstanding, if the goal is to evaluate the effect of SSS techniques, the need for homogeneity in the bone filler is imperative, thus eliminating bias when comparing the superiority of one material against another.

In keeping with this observation, data extraction is made seeking comparability between studies; a fact allowing a meta-analytic approach to assessing the dimensional changes on RW between CM and FGG groups at different cutoffs points of the alveolar bone crest.

**Fig. 5:**
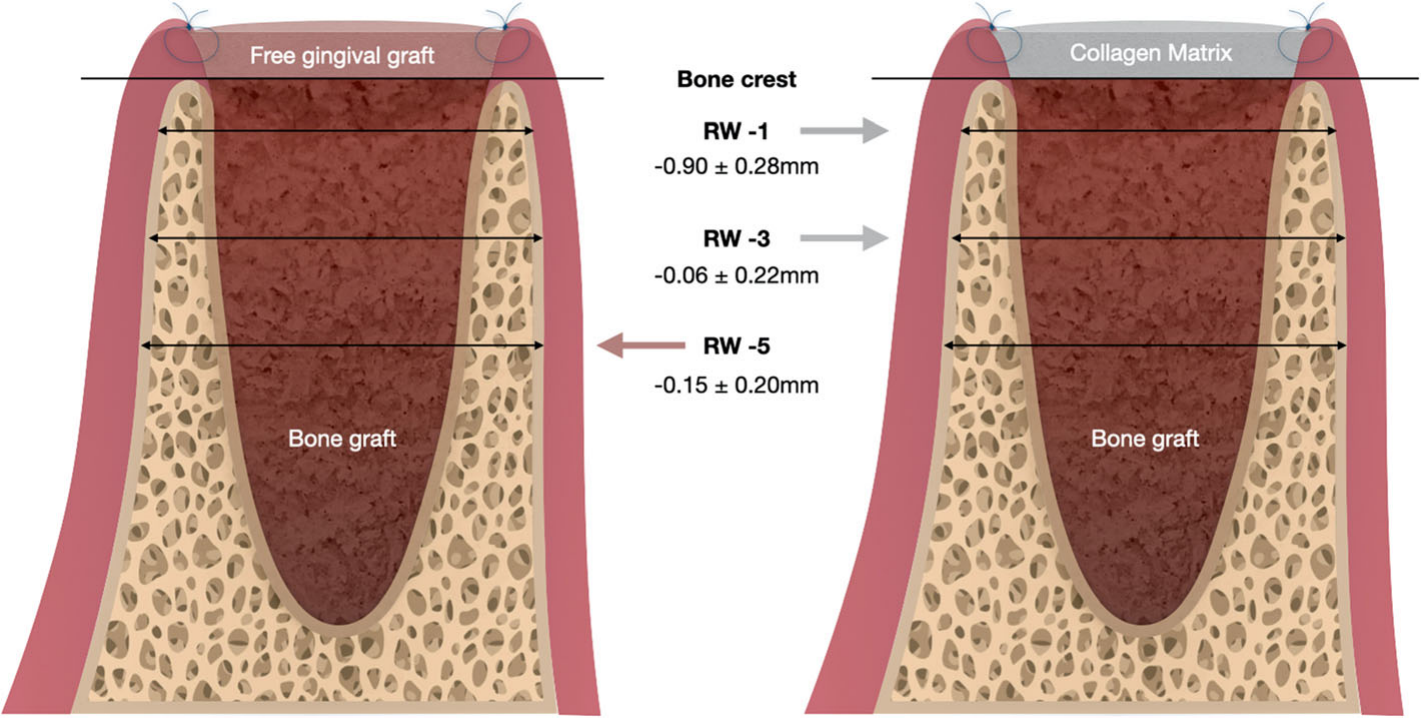
Meta-analytic illustration shows a trend for slight dimensional changes on the ridge width (RW) in the collagen matrix group at − 1 and − 3 mm cutoffs and a slight RW gain of 0.15 mm favoring free gingival graft sealing material

Meta-analyses results did not show significant statistical differences (*p* ≥ 0.05), but a trend for slight dimensional changes on the ridge width in the CM group at − 1 and − 3 mm cutoffs, with mean differences of − 0.09 mm and − 0.06 mm respectively. These findings suggest that use of FGG as socket-sealing material may lessen the alveolar ridge width loss. Moreover, at 5 mm from the bone crest, the differences showed a slight RW gain of 0.15 mm favoring FGG sealing material (Fig. 5). The adequacy for accrued data on meta-evidence was tested for the propensity of type I and type II errors, which often occurs in trials with small sample size, as meta-analysis including few studies with sparse sample size.

After conducting the TSA, it was concluded that the required information size to accept or reject the null hypothesis is far from the available meta-evidence on the study topic up to date. Thus, more studies are warranted to determine which socket-sealing surgery is better for its implementation.

### Agreements and disagreements with previous literature

To our knowledge, this is the first systematic review focused on specifically evaluating the different SSS techniques. The use of FGG as SSS can have the benefit of preserving the height of the buccal bone following tooth extraction according to the study of Karaca et al. [36]. Sockets with spontaneous healing had lost height in the buccal bone (− 1.42 mm) after 3 months; conversely, the height was preserved at sockets using SSS without bone filling (− 0.01 mm). This seems to be an adequate alternative for those cases in which the buccal table is greater than 1 mm, and the use of bone filling may not be imperative [42].

Additional benefits of SSS are the effects on soft tissue conditions by maintaining and increasing the keratinized tissue using FGG or CM. Three studies compared the two techniques [35, 37, 39] and found them to be effective and predictable for preserving the alveolar dimensions and attaining a band of keratinized tissue which at the same time affords benefits on peri-implant health [43, 44]. Nevertheless, the use of a CM reduces the risk of necrosis in the FGG. No significant differences were found between the two groups; however, CM was associated with a significantly lower patient morbidity [45], avoiding graft donor site involvement. Patient comfort should be an important factor in considering the appropriate treatment alternative for the patient [46].

Since ARP is incapable of entirely avoiding the alveolar ridge reduction [5], SSS surgery procedures may optimize esthetic results by limiting the postoperative external contour shrinkage [14]. Schneider et al. [39] evaluated the horizontal volume change in sockets with ARP and SSS as compared with unassisted sockets clinically. Six months after the extraction, they found a reduction on dimensional changes of the buccal contour when performing ARP with the use of FGG or CM as SSS. These results are consistent with Thalmair et al. [40], in which they determined that regardless of the use of bone, soft tissue cover minimizes the contraction of the buccal contour.

The use of CS material was suggested not only to protect the graft materials but also to induce blood clot formation and to stabilize the wound [47]. Collagen dressing materials are preferable due to their inherent properties. The material is a hemostatic agent and possesses the ability to stimulate platelet aggregation and enhance fibrin linkage, which may lead to initial clot formation, stability, and maturation [48]. A limited number of studies evaluated this technique in combination with ARP [17, 37, 49–51]. One included study compared it to the use of CM seal and indicated that CS could form an effective barrier for the bone graft in ARP procedures [37]. They found a reduction in coronal ridge width, vertical buccal bone resorption, and a slight increase in buccal gingival thickness compared to CM. The width of keratinized tissue decreased by an average of 0.08 mm in both groups, which shows minimal loss of tissue. The use of CS could be an alternative; however, despite the benefits, these results should be viewed with caution, due to the small number of patients and studies using this alternative.

Even though, most articles recommend the use of absorbable materials for socket seal; intentionally exposed PTFE membrane at post-extraction sites can predictably lead to an increase of keratinized tissue [52] and the preservation of gingival architecture [21]. Although PTFEm is exposed to the oral cavity, they are not permeable to bacteria. Histological data showed that directly after the removal of the membrane there were no endothelial cells or bacterial contamination, on the contrary, initial granulation tissue was found, so PTFE membranes do not seem to interrupt the healing process of the newly formed tissue [53]. However, despite the benefits, the results of the study by Fotek et al. [34] did not find a statistically significant difference when comparing the width of the keratinized tissue and the thickness of the soft tissue compared to an ADM.

Additional techniques have been found on the literature as the ice cream cone technique using a collagen membrane for socket seal [18, 19], the use of PRF as a socket plug [54], or a saddle connective tissue graft [55]. Although these are also techniques commonly used in clinical practice and according to the previously mentioned studies show favorable results, to date, we have not found randomized clinical trials that, according to our inclusion criteria, allow us to add in these or other techniques in this study.

### Efforts and limitations in the review process

The efforts of the present work rely on the comprehensive literature search, the implementation of critical appraisal of literature methodology and the implementation of quantitative synthesis methods to reduce the propensity of type I error, as the use of a “trial sequential analysis” to determine the adequacy of information size.

Due to seeking the best quality of studies and avoiding bias, only randomized clinical trials were included in this study. Note to mention, the need to find studies comparing SS materials based on the same bone filler between groups had a significant impact on the number of included studies. However, it is important to highlight the importance of this criterion, since having homologous comparative groups will allow us to objectively evaluate the effects of socket seal, which is the main objective of this study.

Among the limitations, the variety of materials, the small number of participants per group, the lack of long-term data, and the lack of homogeneity in particulate bone grafts between studies, are sources of clinical and methodological heterogeneity. Moreover, the sparse data on meta-analysis increased the risk of infraestimating or overestimating the effect size and variance of interventions.

### Generality of the review results and future research

A slight trend for lessening the ridge width loss is observed in favor of the FGG approach, but based on sparse data that did not meet the required information size after a sequential trial analysis. This systematic review supports that socket sealing surgery is a technique that can afford benefits than conventional approaches for alveolar preservation. For this reason, its importance should be underscored, to promote further investigations to enhance the body of the evidence, allowing clinicians the draw of evidence-based reliable recommendations.

## Conclusions

Based on the results of this systematic review, there are several effective SSS techniques to improve the clinical results in the preservation of the alveolar ridge and lessen bone resorption. More clinical studies are required to demonstrate the superiority of one technique over another.

## Supplementary Information

**Additional file 1: Appendix S1**

## Data Availability

Not applicable.

## Abbreviations

SSS: Socket seal surgery
ARP: Alveolar ridge preservation
RCT: Randomized clinical trials
FGG: Free gingival graft
CM: Collagen matrix
CS: Collagen sponge
ADM: Acellular dermal matrix
PTFEm: Polytetrafluoroethylene membrane
RW: Alveolar ridge width
RH: Alveolar ridge height
RIS: Required information size
TSA: Trial sequential analysis package
MD: Mean difference
CI: Confidence intervals
