# Supplementary figures and images for: Socket seal surgery techniques in the esthetic zone: a systematic review with meta-analysis and trial sequential analysis of randomized clinical trials
Source: Int J Implant Dent. 2021 Feb 22;7:13. doi: 10.1186/s40729-021-00294-2 (PMC7897591; doi:10.1186/s40729-021-00294-2)

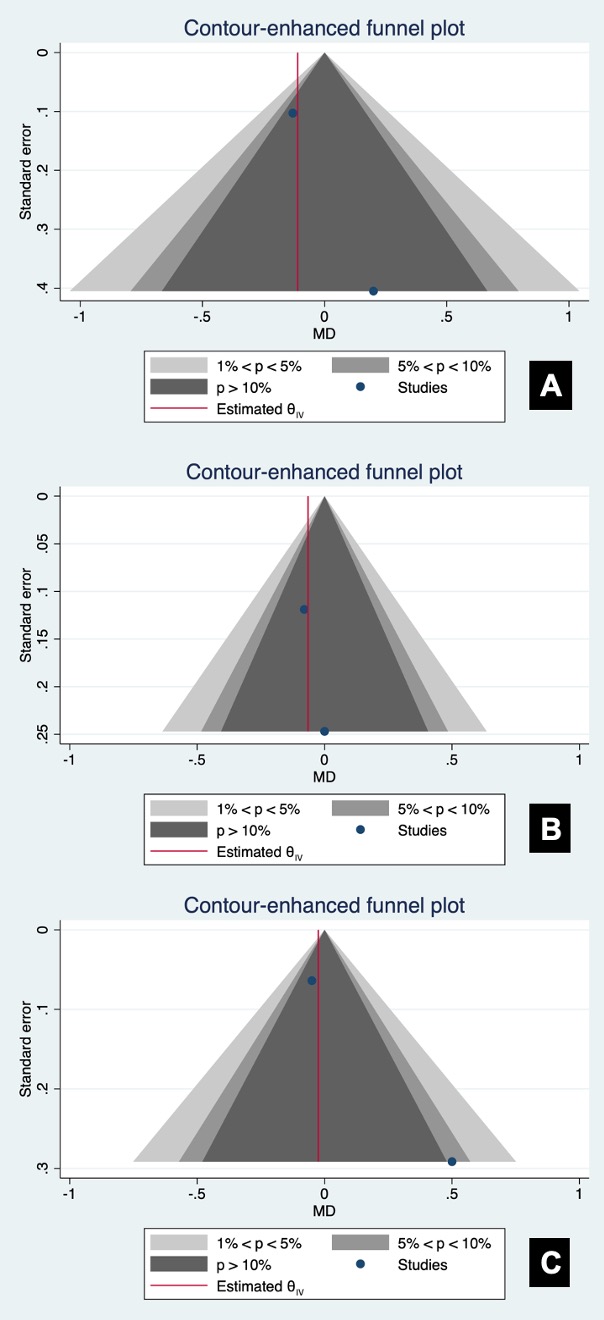

Supplement: Supplementary file 1 — Additional file 1: Appendix S1 [file 40729_2021_294_MOESM1_ESM.jpg]
